# Risk factors associated with severe acute respiratory coronavirus virus 2 (SARS-CoV-2) transmission, outbreak duration, and mortality in acute-care settings

**DOI:** 10.1017/ice.2023.19

**Published:** 2023-10

**Authors:** Tamara R. Duncombe, Matthew Garrod, Xuetao Wang, Joyce Ng, Eunsun Lee, Katy Short, Kennard Tan

**Affiliations:** 1 Infection Prevention and Control, Fraser Health, Surrey, British Columbia, Canada; 2 Department of Pathology and Laboratory Medicine, Fraser Health, Surrey, British Columbia, Canada

## Abstract

**Background:**

Transmission of severe acute respiratory syndrome coronavirus 2 (SARS-CoV-2) in acute-care settings affects patients, healthcare workers, and the healthcare system. We conducted an analysis of risk factors associated with outbreak severity to inform prevention strategies.

**Methods:**

This cross-sectional analysis of COVID-19 outbreaks was conducted at Fraser Health acute-care sites between March 2020 and March 2021. Outbreak severity measures included COVID-19 attack rate, outbreak duration, and 30-day case mortality. Generalized linear models with generalized estimating equations were used for all outcome measures. A P value of 0.05 indicated statistical significance. Analyses were performed using SAS version 3.8 software, R version 4.1.0 software, and Stata version 16.0 software.

**Results:**

Between March 2020 and March 2021, 54 COVID-19 outbreaks were declared in Fraser Health acute-care sites. Overall, a 10% increase in the hand hygiene rate during the outbreak resulted in an 18% decrease in the attack rate (P < .01), 1 fewer death (P = .03), and shorter outbreaks (P < .01). A 10-year increase in unit age was associated with 2.2 additional days of outbreak (P < 0.01) and increases in the attack rate (P < .05) and the number of deaths (P < .01).

**Discussion:**

We observed an inverse relationship between increased hand hygiene compliance during outbreaks and all 3 severity measures. Increased unit age was also associated with increases in each of the severity measures.

**Conclusion:**

This study highlights the importance of hand hygiene practices during an outbreak and the difficulties faced by older facilities, many of which have infrastructural challenges. The latter reinforces the need to incorporate infection control standards into healthcare planning and construction.

The coronavirus disease 2019 (COVID-19) pandemic has largely affected hospitals with increased numbers of patients and increased risk of transmission in a vulnerable population.^1^ Although hospital-acquired COVID-19 only accounts for 6.4% of global cases,^2^ they have a 30-day attributable mortality rate of 16%.^2^ The primary acquisition source for much of these infections is patient-to-patient exposures.^3^ An important tenet of hospital infection prevention and control (IPC) is applying additional precaution measures to those with suspected communicable diseases based on symptoms.^4^ However, transmission of severe acute respiratory coronavirus virus 2 (SARS-CoV-2) often occurs from asymptomatic or presymptomatic cases^5,6^ who would not have the additional precaution measures applied, therefore resulting in transmission to roommates or other contacts. Additionally, in the context of the COVID-19 pandemic, many hospitals often operated at or above capacity, resulting in crowding, further facilitating transmission.^7^

Several strategies have been implemented in hospitals to limit the transmission of SARS-CoV-2 and reduce the impact on unit management.^6^ Commonly used strategies include universal masking, expanded test criteria that include increased testing of symptomatic patients and healthcare workers (HCWs), universal testing of all patients on admission, and designated COVID-19 units.^3^ Other traditional IPC measures, such as standard precautions for all patients, transmission-based precautions for suspected and confirmed cases, and hand hygiene have also been widely practiced.^8^ Because these measures are often implemented simultaneously, determining the efficacy of any one measure is challenging.^3^

Despite best efforts, nosocomial COVID-19 outbreaks occur. Although studies have identified several factors associated with these outbreaks, including shared bathrooms and sinks,^9^ comorbidities of patients involved, and increased staff absence or sickness,^10^ extensive examination of the variety of risk factors that may increase COVID-19 outbreak severity is lacking. Understanding the factors associated with increased outbreak severity can help inform outbreak management. We sought to bridge this gap in knowledge by identifying factors associated with COVID-19 outbreak severity, as measured by attack rate, mortality, and outbreak duration, at hospitals in a large health region of British Columbia, Canada.

## Methodology

### Setting

Fraser Health is the largest regional health authority in British Columbia, Canada.^11^ It provides publicly funded healthcare services to more than 1.9 million people in a geographical area.^12^ The services provided include primary healthcare, community home care, mental health and substance use services, long-term care, and acute medical and surgical services. The setting for this study comprised the 12 hospitals within Fraser Health, with a total of 3,619 acute-care beds across 3 regions (North, South, and East). During the study period, Fraser Health applied multiple strategies to control COVID-19 transmission in hospitals. At the start of the pandemic, Fraser Health implemented universal masking for all HCWs, and in November 2020 Fraser Health implemented universal admission testing of patients. In December 2020, COVID-19 vaccinations became available for HCWs and other eligible populations. During the study period, SARS-CoV-2–positive patients were cohorted on droplet precautions on dedicated units with dedicated staffing. In addition, airborne precautions were applied for all aerosol-generating procedures. All exposed patients were placed on droplet precautions. This study was conducted to support public health and infection control surveillance and was exempt from ethics approval.

### Study design

This study was a retrospective analysis of COVID-19 outbreaks from the 10 hospitals that experienced outbreaks during the study period, March 1, 2020, to March 1, 2021. Data were gathered from various sources at the patient, unit, outbreak, and facility levels. At the patient level, SARS-CoV-2 test results, admission, bed moves, and demographic data were extracted from electronic medical records. Comorbidities, total comorbidity factor and resource intensity weights were obtained from data submitted to the Canadian Institute for Health Information (CIHI). Deaths of outbreak cases were obtained from the public health electronic database.

Facilities management and nursing staff collected facility-level data. Nursing hours were obtained from the employee scheduling system. Each unit’s hand hygiene rates were collected from audit reports for 2 intervals: (1) before the pandemic (March 1, 2019, to March 1, 2020) and (2) during each outbreak. IPC outbreak reports were used to determine outbreak-related measures, including outbreak duration, number of cases, and whether outbreak units were closed, partially opened, or transferred to another unit (moved).

### Case definitions

A confirmed COVID-19 case was a person with laboratory confirmation of SARS-CoV-2 by polymerase chain reaction (PCR). A healthcare-associated (HCA) COVID-19 case was laboratory-confirmed COVID-19 with either (1) symptom onset (or specimen collection date) 5 days or more after admission to a healthcare facility or (2) symptom onset (or specimen collection date) 10 days or less following discharge from a healthcare facility. These criteria were health authority guidelines during the study period. For consistency, the specimen collection date of the first positive test was used as a proxy for symptom onset.

Patients were considered exposed if they had been on the unit in the 2 days preceding the identification of the index case, based on published evidence for COVID-19 infectious period.^13^ Following identification of the index case, all exposed patients were placed on droplet precautions and placed in cohorts separately from any new admissions to the unit, limiting the potential for ongoing transmission from the initial cohort. Patients who tested positive for SARS-CoV-2 in the 60 days before the outbreak were excluded from the exposed patient population for this analysis, according to the health authority’s reinfection definition during the study period.

A COVID-19 outbreak was declared by IPC at the hospital when there was evidence of transmission involving a patient on a unit (as defined by the geographical area, nursing station, and unit mnemonic). Outbreaks were declared in real time as cases were identified. The end of an outbreak was declared 14 days after the last identified exposure to a confirmed case.

Healthcare worker cases and outbreaks occurring on Mental Health and Substance Use (MHSU) units (n = 2) were excluded from this analysis.

### Outcome variables

Outbreak severity was measured for 3 variables: (1) COVID-19 attack rate, defined as the number of new cases divided by the number of exposed patients; (2) outbreak duration, defined as the number of days between outbreak declaration and when the outbreak was declared over; and (3) all-cause 30-day case mortality, defined as the number of outbreak cases who died within 30 days of testing positive for SARS-CoV-2.

### Independent variables and covariates

Independent variables were categorized as patient-level factors (summarized at the outbreak level), unit-level, hospital-level, and outbreak-specific factors. The median of patient-level factors for each outbreak was used for the analysis. A list of all variables and their definitions is included in the Supplementary Material (online).

The number of patient bed moves during the outbreak was included to assess whether patient movement was associated with any outcome variables. Nursing workload and resourcing were measured by the proportion of nursing hours coded as overtime hours and the number of nursing hours per patient day during the outbreak.

Hand hygiene compliance in healthcare workers was assessed by direct observational audits conducted by trained and validated observers. Compliance data were collected electronically using a standardized data collection tool. Audits were conducted monthly in non-outbreak units and daily for units with an outbreak.

### Statistical analysis

Statistical analysis was conducted at the outbreak level. Each risk factor was compared to each of the 3 severity measures using simple linear regression. For model selection, backward elimination and stepwise regression were performed using Akaike information criteria (AIC), and *P* values were used to identify significant independent risk factors. Multivariate Gaussian regression analysis was conducted for attack rate, and multivariate negative binomial analysis was used for the number of deaths and outbreak duration. Generalized estimating equations (GEE) were added as an extension to the models to account for clustering.^14^ Goodness of fit was assessed by analyzing residual plots and deviance residuals. All models included age and sex to control for confounding. A *P* value of .05 was considered statistically significant. Statistical analyses were performed with SAS Studio version 3.8 software,^15^ R Studio version 4.1.0 software,^16^ and Stata/SE version 17.0 software.^17^

## Results

During the study period, 54 outbreaks (Table 1) met the inclusion criteria for analysis, involving 454 confirmed patient cases and 1,516 exposed patients (Table 2). Moreover, 12 outbreaks occurred in hospitals in the East region, 27 in the North region, and 15 in the South region. Among them, 31 outbreaks (57%) occurred in medicine units. The median attack rate was 24% (interquartile range [IQR], 8%–41%). The 30-day case mortality ranged from 0 to 12 deaths, with a median of 1 death (IQR, 0–3). The median outbreak duration was 21 days (IQR, 16–28; range, 6–49). Community incidence during the study period was 8.6 cases per 100,000 population (0.17–32.17 per 100,000), whereas unit attack rates ranged from 2% to 88%.

**Table 1.** Description of Hospitals in the Region With at Least 1 Outbreak During the Study Period

Region/ Hospital	Hospital Type	Hospital Services	Hospital Size, Beds	Teaching Hospital	No. of Outbreaks	Outbreak-Associated Cases
East 1	Regional	Adult and pediatric	257	N	3	7
East 2	Community	Adult and maternity	131	N	2	29
East 3	Community	Adult and pediatric	188	N	4	38
East 4	Community	Adult	45	N	2	36
East 5	Community	Inpatient rehabilitation	25	N	1	10
East totals	…	…	646	0	12	120
North 1	Community	Adult and pediatric, tertiary palliative	259	N	12	125
North 2	Community	Adult	173	N	2	22
North 3	Community	Patient transition to home	24	N	1	3
North 4	Community	Inpatient rehabilitation	96	N	3	19
North 5	Regional	Adult and pediatric, tertiary, specialty cardiac, trauma, neurosurgery	426	Y	4	18
North 6	Community	Adult and maternity	157	N	5	37
North totals	…	…	1,135	1	27	224
South 1	Community	Adult	58	N	1	19
South 2	Community	Adult and maternity	171	N	1	1
South 3	Regional	Adult and pediatric	660	Y	13	90
South totals			889	1	15	110
Region totals			2,670	2	54	454

**Table 2.** Patient-Level Factors of Patients on COVID-19 Outbreak Units

Patient Characteristics	Median (Range)^a^	
	COVID-19 Cases (N=454)	COVID-19 Exposed Patients (N=1,516)
Patient age	79 (18–99)	73 (17–106)
Sex, female, %	50	48
Resource intensity weight	4.56 (0.14–96.97)	1.70 (0.10–72.89)
Length of stay, days	34 (1–813)	12 (1–280)
Comorbidity total factor	2.35 (1.00–100.00)	1.43 (1.00–49.76)
Bed moves within outbreak unit	2 (2–15)	1 (1–3)
Case mix group estimated length of stay	12.2 (1.2–74.6)	7.3 (1.0–75.9)
Chronic flags per patient	3 (0–9)	2 (0–10)

a Unless otherwise indicated.

### Crude analysis

The crude analysis identified 4 patient-level factors that led to increased attack rates: resource intensity weight (8% per unit; 95% confidence interval [CI], 5%–11%), acute length of stay (9% per day; 95% CI, 4%–14%), total comorbidity factor (14% per unit; 95% CI, 5%–23%), and bed moves (38% per move; 95% CI, 20%–55%) (Table 3). The attack rate decreased with an increase in nursing hours per patient day (1% per hour; 95% CI, 0%–2%). Regional hospitals had a 13% (95% CI, 2%–24%) lower attack rate than community hospitals.

**Table 3.** Crude Analysis of Effect of Covariates on Severity Measures Using Simple Linear Regression

Variable	Attack Rate (95% Confidence Interval)			Deaths (95% Confidence Interval)			Outbreak Duration (95% Confidence Interval)		
	Estimate	Lower	Upper	Estimate	Lower	Upper	Estimate	Lower	Upper
**Patient-level factors, median**									
Age, y	0.005	−0.001	0.011	0.049	−0.050	0.148	0.264	−0.025	0.552
Sex, female, %	0.712^a^	0.189	1.235	1.815	−1.053	4.683	−1.082	−27.903	25.740
Resource intensity weight	0.078^b^	0.046	0.110	−0.137	−0.451	0.177	0.722	−1.015	2.459
Length of stay, d	0.007^b^	0.003	0.010	−0.013	−0.052	0.026	0.085	−0.089	0.258
Acute-care length of stay, d	0.009^b^	0.004	0.014	−0.022	−0.069	0.025	0.049	−0.210	0.309
Days between admission and positive test	0.001	−0.002	0.004	−0.009	−0.083	0.065	−0.013	−0.138	0.112
Days between positive test and discharge	0.003	−0.001	0.008	−0.044	−0.102	0.014	0.124	−0.076	0.325
Comorbidities	0.143^b^	0.054	0.232	−0.016	−0.464	0.432	0.909	−3.382	5.200
Bed moves within outbreak unit	0.378^b^	0.204	0.552	0.039	−0.717	0.795	0.878	−10.616	12.371
Case-mix group estimated length of stay	0.008	0.000	0.016	−0.057	−0.169	0.055	0.132	−0.235	0.498
Chronic flags per patient	0.014	−0.056	0.085	−0.175	−0.904	0.554	3.688^a^	0.526	6.850
**Unit-level factors, median**									
Unit age, y	0.002	−0.002	0.006	0.051^a^	0.003	0.099	0.223^b^	0.058	0.388
Sinks in any rooms	−0.075	−0.229	0.079	−0.064	−0.158	0.030	4.838	−2.029	11.705
Overtime nursing hours	−0.379	−1.968	1.210	34.258^b^	16.077	52.439	79.158*	0.820	157.496
Nursing hours per patient day	−0.011^a^	−0.022	0.000	−0.060	−0.342	0.222	−0.433	−0.937	0.070
Hand hygiene rates prior to outbreak	0.846	−0.176	1.868	−3.112	−16.700	10.476	29.987	−16.486	76.459
Hand hygiene rates during outbreak	0.710	−0.371	1.791	−10.018	−27.464	7.428	1.391	−47.457	50.240
Partially opened									
Yes	−0.086	−0.202	0.030	0.298	−2.047	2.643	1.088	−4.256	6.432
Moved	−0.105	−0.284	0.074	−0.386	−1.908	1.136	2.305	−5.941	10.550
No (reference)	…	…	…	…	…	…	…	…	…
Proportion rooms with single beds	−0.004	−0.022	0.014	0.103	−0.125	0.331	−2.891	−13.418	7.636
Ratio of washrooms to beds	0.029	−0.392	0.449	−1.977	−7.286	3.332	−5.461	−24.275	13.354
**Unit type, median**									
Cardiac	−0.255	−0.707	0.198	−1.800	−7.210	3.610	−4.600	−24.803	15.603
Critical care	−0.156	−0.417	0.105	−0.800	−3.924	2.324	1.800	−9.864	13.464
Medicine	−0.014	−0.213	0.185	0.781	−1.599	3.161	4.819	−4.069	13.708
Older adult	0.106	−0.346	0.559	4.200	−1.210	9.610	1.400	−18.803	21.603
Patient transition to home	−0.073	−0.526	0.379	−0.800	−6.210	4.610	17.400	−2.803	37.603
Rehabilitation	−0.002	−0.228	0.225	−1.500	−4.205	1.205	4.600	−5.502	14.702
Surgery (reference)	…	…	…	…	…	…	…	…	…
**Hospital-level factors, median**									
**Hospital type**									
Regional	−0.126^a^	−0.236	−0.016	−0.756	−2.202	0.690	−4.206	−9.245	0.833
Community (reference)	…	…	…	…	…	…	…	…	…
**Region**								0.000	0.000
North	0.093	−0.046	0.232	−2.148^a^	−3.863	−0.433	−3.889	−10.211	2.433
South	−0.011	−0.166	0.143	−1.200	−3.115	0.715	−1.467	−8.524	5.591
East (reference)				…	…	…	…	…	…

a *P*< .05.

b *P* < .01.

**Table 4.** Changes in Severity Measures by Covariates of Interest Using Multiple Linear Regression

Risk Factors	Effect Estimates								
	Attack Rate and 95% Confidence Interval			30-Day Case Mortality and 95% Confidence Interval			Outbreak Duration and 95% Confidence Interval		
	Estimate, Median	Lower	Upper	Estimate, Median	Lower	Upper	Estimate, Median	Lower	Upper
Length of stay	0.006^a^	0.00	0.01				0.016^a^	0.01	0.02
Comorbidity total factor	0.142^a^	0.08	0.21				0.109^b^	0.02	0.20
Hand hygiene rates during outbreak	−1.775^a^	−2.81	−0.74	−10.32^b^	−19.66	−0.99	−5.24^a^	−7.66	−2.82
Hand hygiene rates prior to outbreak							2.68^a^	0.60	5.16
Bed moves	0.468^a^	0.33	0.61						
Unit age	0.003^b^	0.00	0.01	0.04^a^	0.02	0.07	0.018^a^	0.01	0.02
Nursing hours to patient days	0.017^b^	0.00	0.03						
**Facility type**									
Regional	−0.150^b^	−0.27	−0.03				−0.337^b^	−0.62	−0.05
Community (reference)	…	…	…				…	…	…
**Region**									
North	−0.118^b^	−0.21	−0.03	−1.34^a^	−2.35	−0.34	−0.308^a^	−0.45	−0.17
South	−0.084	−0.21	0.04	−0.31	−1.31	0.69	0.23	−0.10	0.55
East (reference)	…	…	…	…	…	…	…	…	…

a *P* < .01.

b *P*< .05.

Unit age and mortality were positively correlated, with 1 death (95% CI, 0.1–2.0) observed for every 20-year increase in unit age (Table 4). Similarly, a 10% increase in nursing overtime hours was associated with ∼3 additional deaths (95% CI, 1.6–5.2). The North region had ∼2 fewer deaths (95% CI, 0.4–3.9) associated with their outbreaks compared to the East region.

Outbreak duration was increased for units with patients who had multiple comorbidities, and each addition to the median number of comorbidities increased outbreak duration by 3.7 days (95% CI, 0.5–6.9). Unit age was also an important factor in outbreak duration; each decade was associated with 2.2 additional days of outbreak (95% CI, 0.6–3.9). Finally, overtime nursing hours were important for duration in the crude models; a 10% increase in overtime hours was associated with 8 additional days of outbreak (95% CI, 0.8–16.0).

### COVID-19 attack rate

After adjusting for age, sex, and other covariates, a 10% increase in the hand hygiene rate during the outbreak resulted in an 18% decrease in the attack rate (*P* < .01). Conversely, a 10-point increase in the median total comorbidity score was associated with a 14% increase in the attack rate (*P* < .01). A 1.4% increase in the attack rate was observed for every 3 bed moves per patient (*P* < .01). Regional hospitals were associated with a lower attack rate than community hospitals even after adjusting for confounders (*P* = .02). Although not statistically significant in the crude analysis, hospitals in the North region appeared to be associated with a lower attack rate than hospitals in the East region (*P* = .01).

### 30-day case mortality

Hand hygiene rates during the outbreak were strongly associated with fewer deaths, and each 10% increase in rates was associated with 1 fewer death (*P* = .03). The North region was protective against severity, with 1.3 fewer deaths occurring on average compared to the East region (*P* < .01).

### Duration

The importance of hand hygiene during the outbreak was demonstrated in the outbreak duration, where each 20% increase was associated with a reduction of 1 day (*P* < .01). Paradoxically, hand hygiene rates in the year prior to the pandemic showed the opposite effect, with increased hand hygiene rates associated with longer outbreaks. Facility factors were once again important, with regional hospitals reducing outbreak duration by ∼8 hours (*P* = .02) and North hospitals reducing outbreak duration by roughly the same (*P* < .01). Patient-level factors played a role in outbreak duration, with each additional comorbidity to the median number increasing duration by 0.11 days (*P* = .02). Length of stay played a role in duration, but the effect was minimal; every 62 days in acute care was associated with an increase of 1 additional day (*P* < .01).

## Discussion

In this study, hand hygiene, facility unit age, and patient comorbidity were associated with COVID-19 outbreak severity within Fraser Health hospitals. A relationship between increased hand hygiene compliance rates during outbreaks and attack rate, 30-day case mortality, and outbreak duration was also observed. Increased hand hygiene compliance during an outbreak provided the largest protective effect compared to other measures. This observation is consistent with literature on the role of hand hygiene in reducing healthcare-associated infections.^18,19^ In contrast, higher hand hygiene compliance rates before the pandemic were associated with higher outbreak severity. Although not directly evaluated, it is possible that historical infection control practices among HCWs did not reflect practices during the COVID-19 pandemic.

An increase in unit age was associated with increases in attack rate, 30-day case mortality, and outbreak duration. Unit age may serve as an indicator of infrastructural challenges. Several studies suggest that private room accommodations and the availability and placement of hand hygiene facilities reduce healthcare-associated infections.^20–23^ Standards for hospital design, and guidelines for managing patients with infectious conditions, have evolved to reflect current evidence.^24,25^ However, older units built before the development of such standards may not be equipped with adequate hand hygiene facilities, single-patient rooms, or dedicated airborne isolation rooms to prevent transmission. Our analysis did not find any association between these individual features and outbreak severity; however, the observed association between unit age and outbreak severity could be due to latent variables or interactions between variables not measured in this study. The association between unit age and COVID-19 outbreaks was corroborated in other studies.^26,27^ One strength of this study is that instead of classifying unit age based on facility age, unit age took into consideration renovation to older facilities with the expectation of compliance with current standards.^24^

The total comorbidity factor is “the cumulative percentage increase on the patient cost associated with all comorbidity codes of the case.”^28^ Using the total comorbidity factor as a proxy for clinical complexity, a 10% increase in this measure was associated with a 10% and 11% increase in attack rate and outbreak duration (respectively). This finding was expected because patients with more complex medical conditions are more susceptible to acquiring infections.^29,30^

Our findings support an inverse correlation between staffing levels and healthcare-associated infections.^31–34^ In the context of COVID-19 transmission, similar findings were observed in long-term care settings.^35,36^ However, in this study, as the ratio of nursing hours to patient days increased, a marginal increase in the attack rate was observed which is inconsistent with studies from non–acute-care settings.^33,34^ Notably, differences in metrics used to quantify hospital staffing, making comparisons between studies challenging.^33,34^ Additionally, we did not measure individual nursing experience, adherence to infection prevention and control practices, or staff skills and abilities that could affect the observed outcome. Future research could examine the impact of these contextual factors to better understand the role of staffing on healthcare-associated COVID-19 transmission. Finally, in the crude analysis, an increase in the proportion of nursing overtime hours was associated with increased outbreak duration and 30-day case mortality. However, these findings were not significant after controlling for covariates. The study’s small sample size may have contributed to the lack of statistical significance.

This study had several other limitations. Summary measures were utilized for patient-level data; thus, the findings cannot be inferred to determine an individual patient’s risk for COVID-19 acquisition in healthcare. Additionally, the study did not include HCW cases because it was not possible to identify all HCW cases associated with each outbreak due to the constraints of self-reporting. The findings of this study may not be easily extrapolated to other hospitals due to differences in practices (eg, universal admission screening for SARS-CoV-2) and surveillance definitions. In addition, this study only applies to the SARS-CoV-2 variants observed during the study period. Other variants may have different infection dynamics and transmissibility.

Increased hand hygiene compliance was observed as one of the major predictors of decreased outbreak severity. Although there are inherent biases within this measure due to the Hawthorne effect,^37^ it is the only method that considers appropriate hand hygiene before, during, and after patient care.^38^ Additionally, direct observations utilizing standardized data collection tools performed by trained and validated observers, as in this study, are considered the gold standard for hand hygiene compliance measurement.^38^

Outbreaks on Mental Health and Substance Use (MHSU) units during the study period were excluded as infection control measures and outbreak management strategies may differ in such settings.^39^ Subsequently, by excluding MHSU units, the findings are not generalizable to that context. The use of specimen collection date as a proxy for symptom onset may have resulted in fewer patients designated as exposed to the index case, which subsequently could lead to an overestimation of the attack rate. Because the proxy was used for all outbreaks, it is unlikely to have biased the results of the analysis.

In this study, we classified patients as susceptible to reinfection after 60 days from their first SARS-CoV-2–positive test, whereas most studies have used 90 days. This feature of our study may have introduced misclassification errors; however, 60 days was applied consistently, minimizing the potential for bias in the results.

Although cases of patients who tested positive within 10 days of discharge from the hospital were considered healthcare associated, given the lengthy incubation period for SARS-CoV-2 (2–14 days),^40^ there was the potential for misclassification errors dependent on community incidence. During the study period, the average community incidence in the region was 8.57 cases per 100,000, resulting in minimal risk of misclassification as healthcare associated.

Finally, patients discharged home from an outbreak unit may have acquired SARS-CoV-2 due to exposure on the unit but were not tested in the community due to clinical presentation or test availability. These unidentified cases may have resulted in underestimation of the attack rate and case mortality but were unlikely to bias the study results in any direction.

The findings of this study, along with the limitations, have identified opportunities for future research, including a similar analysis at the patient level to better understand the individual factors that could lead to excess mortality. Additional risk factors that were not identified at a summary level may be identified by focusing the analysis at an individual level.

## Supplementary material

For supplementary material accompanying this paper visit https://doi.org/10.1017/ice.2023.19. click here to view supplementary material
