# Supplementary material for: Risk factors associated with severe acute respiratory coronavirus virus 2 (SARS-CoV-2) transmission, outbreak duration, and mortality in acute-care settings
Source: Infect Control Hosp Epidemiol. 2023 Feb 23;44(10):1643–9. doi: 10.1017/ice.2023.19 (PMC10587374; doi:10.1017/ice.2023.19)
Supplement: Supplementary file 1 [file S0899823X23000193sup001.docx]

**Supplementary Material**

| **Variable** | **Description** |
| --- | --- |
| **Outcome variables** | |
| *Outbreak attack rate* | The number of new COVID-19 cases associated with the outbreak divided by the number of exposed patients. |
| *Outbreak duration* | The time between outbreak declaration date, typically the date the positive specimen from the first healthcare-associated case resulted, and the date the outbreak was declared over, in days. |
| *All-cause 30-day case mortality* | The number of outbreak-associated COVID-19 cases who died within 30 days of testing positive for SARS-CoV-2. |
| **Independent variables** | |
| ***Patient-level factors*** | |
| *Age* | Patient age in years on admission. |
| *Sex* | Patient sex as recorded in electronic patient health record. |
| *Resource intensity weight (RIW)* | A Canadian Institute of Health Information (CIHI) standardized measure of the intensity of resource use associated with the patient profile based on diagnostic and surgical procedures and patient demographic characteristics. Used as a proxy for clinical complexity. |
| *Length of stay* | Total patient hospital length of stay in days. |
| *Acute length of stay* | Patient hospital length of stay excluding days registered as alternate level of care, defined as medically fit for discharge but cannot access the appropriate post-acute care services. |
| *Days between admission and positive specimen collection* | Time between hospital admission date and date of positive specimen collection in days. |
| *Days between positive specimen collection and discharge* | Time between positive specimen collection date and hospital discharge date in days. |
| *Comorbidity total factor* | A CIHI standardized measure of the cumulative percentage increase in patient cost associated with all comorbidity codes for the patient. Used as a proxy for clinical complexity. |
| *Case mix group estimated length of stay* | A CIHI standardized measure of the average acute length of stay in hospital for patients with similar clinical and demographic profiles. . Used as a proxy for clinical complexity. |
| *Bed moves* | Number of bed moves for the patient within the outbreak unit during the outbreak. |
| *Number of chronic flags* | The count of patient chronic diseases calculated from comorbidities ICD-10-CA codes extracted from coded discharge data submitted to CIHI from patient medical records. |
| ***Unit*** | |
| *Unit age* | The number of years since the unit space was built or the last major renovation to the space, whichever is most recent. |
| *Any rooms with hand hygiene sinks* | A dichotomous (yes or no) variable indicating whether patient rooms on the unit have dedicated hand hygiene sinks. |
| *Nursing hours coded as overtime (OT) hours during the outbreak* | The proportion of total nursing hours during the outbreak that were coded as overtime hours. Used as a measure of nursing workload. |
| *Number of nursing hours divided by the number of patient days during the study period* | The total number of nursing hours divided by the number of patient days for the unit during the outbreak. Used as a measure of nursing workload. |
| *Hand hygiene compliance rates prior to the outbreak* | The average hand hygiene compliance audit result for the unit in the year prior to the COVID-19 pandemic (March 1, 2019 to March 1, 2020). |
| *Hand hygiene rates during outbreak* | The average hand hygiene compliance audit result for the unit during the outbreak. |
| *Unit partially open* | Categorical variable indicating whether the unit was opened to new patient admissions during the outbreak, with the exposed patients and confirmed cases cohorted respectively, or whether the cohorted exposed patients were moved to a different unit. |
| *Proportion of patient rooms with single beds* | The proportion of private patient rooms on the unit. |
| *Ratio of patient washrooms to beds* | The number of patient washrooms on the unit divided by the number of patient beds. |
| *Unit type* | Unit service type. |
| ***Hospital*** | |
| *Hospital type* | Dichotomous variable of hospital type, regional or community. |
| *Region* | Categorical variable based on physical location of hospital within Fraser Health service area (North, South, East). |
